# A Hospice Transitions Program for Patients in the Emergency Department

**DOI:** 10.1001/jamanetworkopen.2024.20695

**Published:** 2024-07-08

**Authors:** Christopher W. Baugh, Kei Ouchi, Jason K. Bowman, Ayal A. Aizer, Alexander W. Zirulnik, Martha Wadleigh, Angela Wise, Paula Remón Baranda, Richard E. Leiter, Bradley J. Molyneaux, Andrea McCabe, Panupong Hansrivijit, Kate Lally, Melissa Littlefield, Alexei M. Wagner, Katherine H. Walker, Hojjat Salmasian, Kourosh Ravvaz, Jada A. Devlin, Karen Lewis Brownell, Matthew P. Vitale, Frantzie C. Firmin, Nelia Jain, Jane deLima Thomas, James A. Tulsky, Soumi Ray, Lynne M. O’Mara, Elizabeth M. Rickerson, Mallika L. Mendu

**Affiliations:** 1 Department of Emergency Medicine, Brigham and Women’s Hospital, Boston, Massachusetts; 2 Department of Psychosocial Oncology and Palliative Care, Dana Farber Cancer Institute, Boston, Massachusetts; 3 Department of Radiation Oncology, Brigham and Women’s Hospital, Boston, Massachusetts; 4 Department of Medical Oncology, Dana Farber Cancer Institute, Boston, Massachusetts; 5 Massachusetts Department of Public Health, Boston, Massachusetts; 6 Dartmouth Engineering Thayer School, Hanover, New Hampshire; 7 Department of Medicine, Brigham and Women’s Hospital, Boston, Massachusetts; 8 Department of Neurology, Brigham and Women’s Hospital, Boston, Massachusetts; 9 Mass General Brigham Home Hospital, Boston, Massachusetts; 10 Division of Renal Medicine, Brigham and Women’s Hospital, Boston, Massachusetts; 11 Division of Pharmacoepidemiology and Pharmacoeconomics, Brigham and Women’s Hospital, Boston, Massachusetts; 12 Office of the Chief Operating Officer, Brigham and Women’s Hospital, Boston, Massachusetts; 13 Department of Analytics, Planning, Strategy and Improvement, Brigham and Women’s Hospital, Boston, Massachusetts; 14 Department of Surgery, Brigham and Women’s Hospital, Boston, Massachusetts; 15 Department of Anesthesiology, Perioperative and Pain Medicine, Brigham and Women’s Hospital, Boston, Massachusetts

## Abstract

**Importance:**

Patients often visit the emergency department (ED) near the end of life. Their common disposition is inpatient hospital admission, which can result in a delayed transition to hospice care and, ultimately, an inpatient hospital death that may be misaligned with their goals of care.

**Objective:**

To assess the association of hospice use with a novel multidisciplinary hospice program to rapidly identify and enroll eligible patients presenting to the ED near end of life.

**Design, Setting, and Participants:**

This pre-post quality improvement study of a novel, multifaceted care transitions program involving a formalized pathway with email alerts, clinician training, hospice vendor expansion, metric creation, and data tracking was conducted at a large, urban tertiary care academic medical center affiliated with a comprehensive cancer center among adult patients presenting to the ED near the end of life. The control period before program launch was from September 1, 2018, to January 31, 2020, and the intervention period after program launch was from August 1, 2021, to December 31, 2022.

**Main Outcome and Measures:**

The primary outcome was a transition to hospice without hospital admission and/or hospice admission within 96 hours of the ED visit. Secondary outcomes included length of stay and in-hospital mortality.

**Results:**

This study included 270 patients (median age, 74.0 years [IQR, 62.0-85.0 years]; 133 of 270 women [49.3%]) in the control period, and 388 patients (median age, 73.0 years [IQR, 60.0-84.0 years]; 208 of 388 women [53.6%]) in the intervention period, identified as eligible for hospice transition within 96 hours of ED arrival. In the control period, 61 patients (22.6%) achieved the primary outcome compared with 210 patients (54.1%) in the intervention period (*P* < .001). The intervention was associated with the primary outcome after adjustment for age, race and ethnicity, primary payer, Charlson Comorbidity Index, and presence of a Medical Order for Life-Sustaining Treatment (MOLST) (adjusted odds ratio, 5.02; 95% CI, 3.17-7.94). In addition, the presence of a MOLST was independently associated with hospice transition across all groups (adjusted odds ratio, 1.88; 95% CI, 1.18-2.99). There was no significant difference between the control and intervention periods in inpatient length of stay (median, 2.0 days [IQR, 1.1-3.0 days] vs 1.9 days [IQR, 1.1-3.0 days]; *P* = .84), but in-hospital mortality was lower in the intervention period (48.5% [188 of 388] vs 64.4% [174 of 270]; *P* < .001).

**Conclusions and Relevance:**

In this quality improvement study, a multidisciplinary program to facilitate ED patient transitions was associated with hospice use. Further investigation is needed to examine the generalizability and sustainability of the program.

## Key Points

**Question:**

Is a multidisciplinary program aimed at the timely identification and transition of eligible patients presenting near the end of life to the emergency department associated with a significant increase in goal-concordant hospice care?

**Findings:**

In this quality improvement study, 61 of 270 patients (22.6%) achieved the primary outcome of goal-concordant transition to hospice within 96 hours in the control period compared with 210 of 388 (54.1%) in the intervention period. In addition, the presence of a Medical Order for Life-Sustaining Treatment was independently associated with hospice transition across all groups (adjusted odds ratio, 1.88; 95% CI, 1.18-2.99).

**Meaning:**

Hospitals can investigate the implementation of a similar hospice transition program to improve use of hospice at their institutions.

## Teaser

This quality improvement study evaluates the association of hospice use with a novel multidisciplinary hospice program to rapidly identify and enroll eligible patients presenting to the emergency department near the end of life.

## Introduction

Patient preference and guideline-based care support hospice use at the end of life.^1,2,3^ Hospice care is associated with improved symptoms and caregiver outcomes.^4,5^ Traditionally, hospice care has occurred at home, but use of general inpatient (GIP) hospice has increased.^6^ Inpatient hospice requires 1 or more transitions, and timely patient identification and transition logistics pose challenges.^1,7,8^ Processes of care that anticipate clinical needs, involving interdisciplinary teams, can provide a seamless transition to hospice care.

There are myriad challenges associated with transitioning patients to hospice after emergency department (ED) arrival, including lack of clarity regarding goals of care, hospice access, and sporadic arrival of patients appropriate for transition to hospice.^9^ Without a defined clinical pathway, similar to other emergency conditions with well-established workflows (such as acute stroke), operationalizing timely hospice care is challenging. The decision to admit a patient near the end of life to an inpatient hospital service, as opposed to transitioning to hospice care, is multifactorial, including patient and family acceptance as well as knowledge gaps regarding the benefits of hospice care (eg, nursing availability 24 hours a day and 7 days a week, access to social workers and chaplains, and bereavement services).^10^

Despite the potential benefits to patients and the health care system, historically, few patients transition directly from the ED to hospice. Efforts to identify ED patients benefiting from hospice referral have been limited. Liberman et al^11^ conducted a pre-post observational study with 82 ED patients, in which intervention patients received goals of care discussions and, if appropriate, home hospice referral; 39.3% of patients in the intervention cohort were referred to home hospice compared with 0% in the control cohort. Previous efforts to boost hospice enrollment after ED presentation have demonstrated promising results with small cohorts or patients with specific conditions (eg, cancer), but larger, more generalized experiences have been limited.^12,13,14^ We describe the design and implementation of a care transitions program (CTP) with the primary objective of improving the timeliness of goal-concordant hospice care, for patients identified by their care team to be near end of life and eligible for hospice.

## Methods

### Participants and Exposure

**Figure 1.**
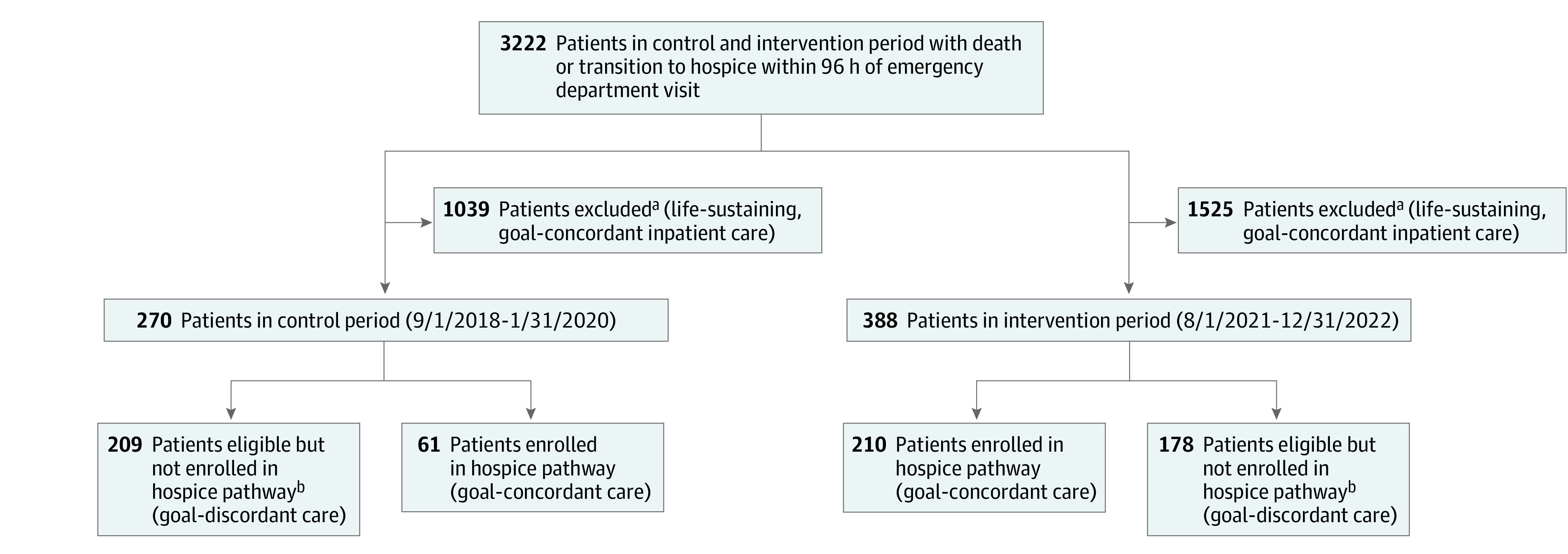
Patient Cohort Selection ^a^Excluded if the patient’s goals were inconsistent with hospice care based on electronic health record review; specific criteria included inpatient admission for surgery, procedure, radiation therapy, or aggressive resuscitative measure (such as endotracheal intubation, vasopressor therapy, central line placement, or noninvasive positive pressure ventilation). ^b^Eligible but not enrolled in hospice pathway if there was an inpatient hospital admission followed by death within 96 hours without the use of hospice, despite an intention to transition to end-of-life care and comfort-focused care without intensive life-sustaining treatments.

Brigham and Women’s Hospital is a 793-bed, tertiary care academic hospital in Boston, Massachusetts, affiliated with the Dana-Farber Cancer Institute and Harvard Medical School. On average, 1100 inpatient deaths occur annually at Brigham and Women’s Hospital (with an observed mortality rate of 1.9%). Our study population included ED patients 18 years or older presenting with a life-threatening condition resulting in an in-hospital death or transition to hospice within 96 hours of arrival (Figure 1). The control period before the ED CTP was from September 1, 2018, to January 31, 2020. Given the COVID-19 pandemic and to account for an initial 6-month startup period, we excluded February 1, 2020, to July 31, 2021, from the analysis. The intervention period was from August 1, 2021, to December 31, 2022. The Mass General Brigham institutional review board deemed this study as an exempt quality improvement project, which granted a waiver of informed consent to access patient records for data collection. This study followed the Standards for Quality Improvement Reporting Excellence (SQUIRE) reporting guideline.

### Study Design

**Figure 2.**
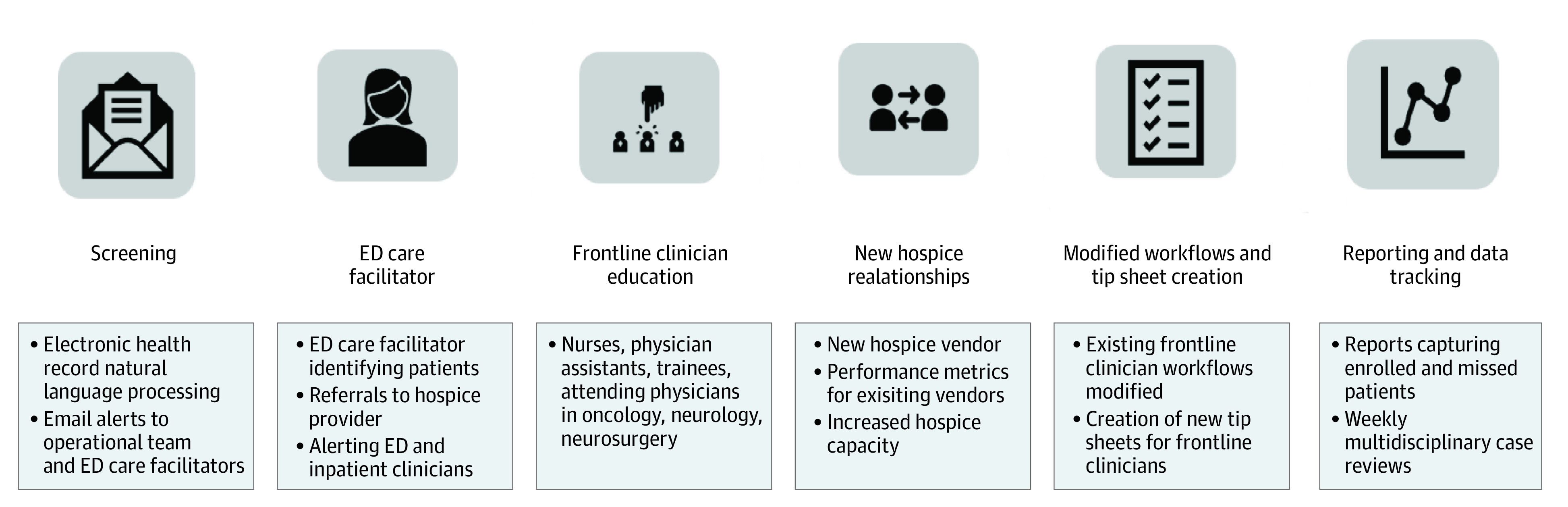
Emergency Department (ED) Care Transitions Program Key Elements

Using a pre-post quality improvement study design, we analyzed data collected prospectively during the implementation of a novel hospice transitions program (ED CTP). We designed and undertook operational planning of ED CTP as a quality improvement initiative from February 1, 2020, to February 28, 2021. A multidisciplinary task force comprising physicians, physician assistants, nurses, social workers, ED care coordinators, palliative care staff, specialty service staff, and data scientists collaboratively developed workflows illustrated in Figure 2 and eFigure 1 in Supplement 1.

### Program Implementation

Key features of the program included screening potential candidates using natural language processing on near–real-time data from the electronic health record (EHR) to alert ED care coordinators via email (eMethods in Supplement 1). The algorithm was a simple string-matching approach using either exact phrases or regular expressions to identify diagnosis codes (neurologic and cancer based) from the admission-discharge-transfer EHR feed, written using Scala programming language. We also introduced dedicated rounding to identify candidates early, as well as biannual virtual education of ED clinicians (eg, nurses, physician assistants, trainees, and attending physicians) about program benefits that featured patient vignettes of patient transitions from the ED to hospice. The decision to pursue GIP hospice was multidisciplinary, led primarily by the ED clinician. The ED care facilitator and hospice partner determined whether the patient was eligible for GIP hospice and, if not eligible, ensured palliative care consultation. The program also included (1) education of specialists (neurosciences and oncology) inclusive of biannual virtual sessions on hospice care offerings, benefits to patients and families, and patient vignettes; (2) point-of-care tip sheets (eFigure 2 in Supplement 1); (3) optimization of hospice admission orders; (4) creation of data reporting, including tracking of eligible patients not enrolled in the hospice pathway; (5) review of patient out-of-pocket costs to ensure observation use and/or hospice did not increase direct expenses; and (6) weekly multidisciplinary case reviews. In addition, at the program outset, we aimed to expand inpatient hospice availability by (1) interviewing multiple hospice vendors and selecting a vendor best able to meet quality and operational needs; (2) renegotiating a contract with an existing hospice vendor that included novel operational and quality metrics; and (3) meeting with vendors monthly to review performance.

### Study Protocol

Data reports were created using our institution’s Enterprise Data Warehouse (EDW), which aggregates data from the EHR to identify potential study participants with transition to hospice and/or in-hospital mortality within 96 hours of ED admission. Patient demographic characteristics (eg, age, sex, race and ethnicity, language, educational level, and primary payer), length of stay (LOS), documentation of prior serious illness conversations, and Charlson Comorbidity Index (CCI) were extracted from the EDW. Data on race and ethnicity (Asian, Black, White, and other [including American Indian or Alaskan Native, ≥2 races or ethnicities, and other; categories were based on categories available in the EHR]) were collected by registration staff via patient self-reporting and added to the EHR. Race and ethnicity have previously been associated with disparate hospice use.^15,16^ Trained research associates (A.W.Z., P.R.B., and P.H.) performed EHR-based record reviews to collect additional key study elements, including variables such as the presence of a Medical Order for Life-Sustaining Treatment (MOLST), code status, diagnoses, and, if applicable, cancer history. We defined patients as “hospice pathway enrolled” if they either died in observation status (not inpatient hospital admission status) in the process of being transitioned to hospice or they transitioned to GIP or home hospice within 96 hours of ED arrival. We defined patients as “eligible but not hospice pathway enrolled” if there was an inpatient hospital admission followed by death within 96 hours without the use of hospice, despite an intention to transition to end-of-life care and comfort-focused care without intensive life-sustaining treatments. Finally, we classified patients as “excluded” if the patient’s goals appeared to be inconsistent with hospice care; life-sustaining, goal-concordant inpatient hospital care criteria included inpatient hospital admission for a procedure such as surgery, procedure, radiation therapy, or aggressive resuscitative measure (such as endotracheal intubation, vasopressor therapy, central line placement, or noninvasivpositive pressure ventilation).

### Outcomes

The primary outcome measure was a planned transition to hospice without inpatient hospital admission within 96 hours of the ED visit, which included patients in observation status in the process of hospice enrollment at their time of death. Secondary outcomes included hospice type, inpatient and ED LOS, and in-hospital mortality (death within the hospital regardless of patient class, such as observation, inpatient, or hospice). We also examined differences between subgroups with cancer and neurologic conditions.

### Statistical Analysis

Categorical baseline covariates were compared using the Fisher exact test. Normally and nonnormally distributed continuous covariates were compared using the *t* test and the Wilcoxon rank sum test, respectively. We used univariable and multivariable logistic regression to identify factors associated with our primary outcome. The key independent variable of interest was the time period before (control) and after (intervention). Models were adjusted for age, insurance status, CCI, and whether a MOLST was completed as well as race and ethnicity. An interaction analysis was used to examine whether a differential association was seen between the primary outcome and enrollments between patients admitted to oncology compared with neurosciences services. The threshold for statistical significance was a 2-sided *P* < .05. Analyses were conducted in SAS, version 9.4 (SAS Institute Inc).

## Results

### Patient Characteristics

Figure 1 illustrates the patient selection strategy; 2564 patients were excluded based on goal-concordant inpatient care. The study included 658 patients: 270 patients (median age, 74.0 years [IQR, 62.0-85.0 years]; 133 of 270 female [49.3%] and 122 of 270 male [45.2%]; 6 of 270 Asian patients [2.2%], 33 of 270 Black patients [12.2%], 183 of 270 White patients [67.8%], and 11 of 270 patients [4.1%] of other race or ethnicity) in the control period and 388 patients (median age, 73.0 years [IQR, 60.0-84.0 years]; 208 of 381 female [54.6%] and 173 of 381 male [45.4%]; 22 of 362 Asian patients [6.1%], 35 of 362 Black patients [9.7%], 281 of 362 White patients [77.6%], and 24 of 362 patients [6.6%] of other race or ethnicity) in the intervention period (Table 1). There were no statistically significant differences between the control period and the intervention period in demographic characteristics, except for primary payer (control: Medicare, 151 of 270 [55.9%]; and commercial, 98 of 270 [36.3%] vs intervention: Medicare, 177 of 388 [45.6%]; and commercial, 190 of 388 [49.0%]; *P* = .006) and MOLST at ED arrival (65 of 270 [24.1%] vs 152 of 388 [39.2%]; *P* = .01).

**Table 1.** Patient Characteristics

Patient characteristic	Control (n = 270)	Intervention (n = 388)	*P* value
Age, median (IQR), y	74.0 (62.0-85.0)	73.0 (60.0-84.0)	.76
Sex, No. (%)^a^			
Male	122 (45.2)	173 (44.6)	.57
Female	133 (49.3)	208 (53.6)	
Race, No. (%)			
Asian	6 (2.2)	22 (5.7)	.07
Black	33 (12.2)	35 (9.0)	
White	183 (67.8)	281 (72.4)	
Other^b^	11 (4.1)	24 (6.2)	
Ethnicity, No. (%)^c^			
Hispanic	16 (5.9)	25 (6.4)	>.99
Non-Hispanic	213 (78.9)	334 (86.1)	
Language, No. (%)^d^			
English	228 (84.4)	333 (85.8)	.63
Spanish	2 (0.7)	6 (1.5)	
Other	26 (9.6)	43 (11.1)	
Educational level, No. (%)^e^			
Graduate school	15 (5.6)	42 (10.8)	.37
College	75 (27.8)	125 (32.2)	
High school	86 (31.9)	129 (33.2)	
Less than high school	27 (10.0)	37 (9.5)	
Other	5 (1.9)	9 (2.2)	
Primary payer, No. (%)			
Medicare	151 (55.9)	177 (45.6)	.006
Commercial insurance	98 (36.3)	190 (49.0)	
Medicaid	12 (4.4)	16 (4.1)	
Other	9 (3.3)	5 (1.3)	
Documentation of prior serious illness conversation, No. (%)	25 (9.3)	47 (12.1)	.26
Presence of a MOLST at the time of ED arrival, No. (%)^f^	65 (24.1)	152 (39.2)	.01
Charlson Comorbidity Index, median (IQR)	4.0 (2.0-4.0)	4.0 (2.0-4.0)	.94
ED LOS, median (IQR), h	3.8 (2.4-6.0)	4.5 (1.8-6.9)	.56
Hospital LOS, median (IQR), d	2.0 (1.0-3.0)	2.0 (1.0-3.0)	.84
In-hospital mortality, No. (%)	174 (64.4)	188 (48.4)	<.001

Abbreviations: ED, emergency department; LOS, length of stay; MOLST, Medical Order for Life-Sustaining Treatment.

^a^ In the control cohort, 15 patients were classified as other (missing, nondefined, or nonbinary), and in intervention cohort, 7 patients were classified as other.

^b^ Includes American Indian or Alaskan Native, declined to answer, 2 or more races, or other race; does not include patients with missing, unknown, or undefined race (37 patients in the control cohort and 26 patients in the intervention cohort).

^c^ Does not include patients with missing, unknown, or undefined ethnicity (41 patients in the control cohort and 29 patients in the intervention cohort).

^d^ Does not include patients with missing, unknown, or undefined language (14 patients in the control cohort and 6 patients in the intervention cohort).

^e^ Does not include patients with missing, unknown, or undefined education (62 patients in the control cohort and 46 patients in intervention cohort).

^f^ Does not include patients with missing MOLST information (130 patients in the control cohort and 133 patients in the intervention cohort).

### Primary and Secondary Outcomes

In the control period, 61 patients (22.6%) were enrolled in hospice or were in observation status in the process of hospice enrollment within 96 hours of the ED visit compared with 210 patients (54.1%) in the intervention period (*P* < .001). Univariable logistic regression showed that the intervention was associated with an increased odds of the primary outcome (odds ratio [OR], 4.04; 95% CI, 2.85-5.73), as was the presence of a MOLST (OR, 2.04; 95% CI, 1.34-3.12) and higher CCI score (OR, 1.12; 95% CI, 1.02-1.23) (Table 2). After adjustment in a multivariable logistic regression model for age, race and ethnicity, insurance, CCI, and presence of a MOLST, the intervention was associated with the primary outcome (OR, 5.02; 95% CI, 3.17-7.94). The presence of a MOLST was also independently associated with the primary outcome after adjustment (OR, 1.88; 95% CI, 1.18-2.99).

**Table 2.** Univariable and Multivariable Logistic Regression Models for Primary Outcome of Patients Transitioning From the Emergency Department to Hospice Within 96 Hours Without Admission

Characteristic	Univariable model, OR (95% CI)	Multivariable model, AOR (95% CI)
Cohort		
Control	1 [Reference]	1 [Reference]
Intervention	4.04 (2.85-5.73)	5.02 (3.17-7.94)
Demographic		
Age (per 1-y increase)	1.00 (0.99-1.02)	1.00 (0.98-1.02)
Race		
Asian	1.39 (0.65-2.99)	1.56 (0.51-4.73)
Black	0.71 (0.42-1.22)	0.62 (0.30-1.28)
White	1 [Reference]	1 [Reference]
Other	0.93 (0.46-1.87)	0.82 (0.30-2.29)
Primary payer		
Commercial insurance	1 [Reference]	1 [Reference]
Medicare	0.76 (0.55-1.04)	0.80 (0.48-1.33)
Medicaid	0.56 (0.25-1.28)	0.87 (0.27-2.76)
Other	0.20 (0.04-0.90)	0.84 (0.12-5.75)
MOLST status		
No MOLST	1 [Reference]	1 [Reference]
MOLST	2.04 (1.34-3.12)	1.88 (1.18-2.99)
Charlson Comorbidity Index	1.12 (1.02-1.23)	1.06 (0.89-1.26)

Abbreviations: AOR, adjusted odds ratio; MOLST, Medical Order for Life-Sustaining Treatment; OR, odds ratio.

Table 3 illustrates hospice type between control and intervention cohorts among patients who achieved the primary outcome. There was a statistically significant difference in hospice type between the cohorts; in the control group, 8 of 61 patients (13.1%) transitioned to GIP hospice, 34 of 61 (55.7%) transitioned to home hospice, 1 of 61 (1.6%) transitioned to an outpatient hospice facility, and 18 of 61 (29.5%) were in the process of transitioning into hospice but died prior to enrollment. In the intervention group, 86 of 210 patients (41.0%) transitioned to GIP hospice, 99 of 210 (47.1%) transitioned to home hospice, 5 of 210 (2.4%) transitioned to an outpatient hospice facility, and 20 of 210 (9.5%) were in the process of transitioning into hospice but died prior to enrollment (*P* < .001). There was no statistically significant difference in inpatient LOS between the control and intervention cohorts (median, 2.0 days [IQR, 1.1-3.0 days] vs 1.9 days [IQR, 1.1-3.0 days]; *P* = .84) (Table 1). There was also no statistically significant difference in ED LOS between the control and intervention cohorts (median, 3.8 hours [IQR, 2.4-6.0 hours] vs 4.5 hours [IQR, 1.8-6.9]; *P* = .56). We observed lower in-hospital mortality in the intervention cohort than in the control cohort (48.5% [188 of 388] vs 64.4% [174 of 270]; *P* < .001).

**Table 3.** Type of Hospice Among Patients Transitioning Within 96 Hours of Emergency Department Admission

Hospice type	No. (%)		*P* value
	Control period (n = 61)	Intervention period (n = 210)	
General inpatient hospice	8 (13.1)	86 (41.0)	<.001
Home hospice	34 (55.7)	99 (47.1)	
Outpatient hospice facility	1 (1.6)	5 (2.4)	
None^a^	18 (29.5)	20 (9.5)	

^a^ Patients in observation status in the process of transitioning into hospice but who died prior to hospice enrollment.

### Subgroup Analysis

eTables 1 and 2 in Supplement 1 describe patients with cancer and neurological diagnoses, respectively. The largest subgroup was patients with cancer, representing 195 of 658 patients (29.6%), followed by patients with neurologic diagnoses, comprising 127 of 658 patients (19.3%). Similar to the overall cohort, there was a significant difference between the control and intervention groups in the presence of a MOLST in the subgroup with neurologic diagnoses (6 of 56 [10.7%] vs 25 of 71 [35.2%]; *P* = .003; eTable 2 in Supplement 1) but not in the subgroup with cancer (25 of 61 [41.0%] vs 74 of 134 [55.2%]; *P* = .09; eTable 1 in Supplement 1). There was a significant difference in prior documented serious illness conversation in the subgroup with cancer (4 of 61 [6.6%] vs 31 of 134 [23.1%]; *P* = .005).

In the subgroup with cancer during the control period, 21 of 61 patients (34.4%) achieved the primary outcome compared with 88 of 134 patients (65.7%) in the intervention period (*P* < .001). The intervention remained associated with the primary outcome after multivariable regression (OR, 4.19; 95% CI, 2.04-8.60; eTable 3 in Supplement 1).

In the subgroup of patients with neurologic diseases during the control period, 18 of 56 patients (32.1%) achieved the primary outcome measure vs 42 of 71 patients (59.2%) in the intervention period (*P* = .004). The association between the intervention and the primary outcome was no longer significant after multivariable regression (OR, 2.05; 95% CI, 0.78-5.37; *P* = .14); however, a MOLST continued to be strongly associated with the primary outcome (OR, 4.36; 95% CI, 1.22-15.55; *P* = .02) (eTable 4 in Supplement 1). The most common diagnoses in this subgroup were hemorrhagic stroke, ischemic stroke, and brain mass. No significant interaction between the primary outcome and being eligible but not hospice pathway enrolled was seen among patients admitted to oncology compared with neurologic services (OR, 1.25; 95% CI, 0.38-4.17; *P* = .71).

## Discussion

In this study performed in a large tertiary care academic hospital affiliated with a cancer center, we found that a collaboratively developed pathway to identify patients in the ED who were near the end of life with goals of care consistent with hospice use was associated with timely hospice use. We also found that the presence of a MOLST was associated with hospice transition, particularly among patients with neurologic diagnoses. Finally, we observed a decrease in in-hospital mortality among patients in the intervention cohort. Key program elements included multidisciplinary involvement in design and operationalization; consistent, iterative education for frontline clinicians; enhanced access to GIP hospice; and program governance to regularly review data to identify improvement opportunities.

Both this study and the study by Liberman et al^11^ illustrate that alternative pathways for avoiding unnecessary admissions for patients near the end of life are feasible and associated with increased hospice use. Critical differences in our approach included a larger cohort, inclusion of nearer-term end-of-life events, expansion of hospice access, and logistical changes to hospice admission. Patients with cancer represented the largest subgroup of those enrolled in our hospice transition program. Nationally, approximately 4.2% of adult ED visits are cancer related, and inpatient hospital admission among these patients is nearly 3 times higher than the overall population.^17,18,19^ Hospice care for patients with poor-prognosis cancers is associated with improved quality of life, decreased hospitalization, and decreased expenditure over the last year of life.^20,21^ Lack of timely recognition and delayed referral to hospice programs can lead to excess inpatient deaths.^22,23,24,25^

Our analysis highlights the benefit associated with a MOLST in facilitating goal-concordant hospice transition, which is consistent with prior evidence.^26,27^ We postulate that the presence of a MOLST may be most influential in guiding surrogate decision-making related to hospice for patients with sudden, catastrophic illnesses. The intervention’s lack of significance on the primary outcome after multivariable regression in the subgroup with neurologic diagnoses may have been due to the smaller sample (type II error).

This study highlights several barriers to implementing and sustaining an ED CTP across a broad patient cohort. First, the timely identification of eligible patients and the logistics of transition are major program elements that must be optimized. We partially addressed this using natural language processing that processed near–real-time data from the EHR. Traditionally, the pathway toward inpatient hospital admission is well resourced, but the pathway toward hospice care is not. Second, expanding inpatient hospice availability through an additional vendor was important.^28,29^ The meaningful 10-fold increase in GIP hospice use between the control and intervention cohorts reflects the crucial role of increasing hospice capacity. Third, in the ED setting, there is often a lack of clarity of goals of care and skills to hold conversations about goals of care effectively.^30^ Both patients and clinicians manage the tension between the desire to explore all treatment options while maintaining quality of life and accepting death with comfort and dignity. Our program has aimed to address knowledge gaps regarding hospice benefits with regular, iterative education. Fourth, given that patients who are eligible for hospice may present infrequently or sporadically (ie, <1 patient daily), hospice is an uncommon ED disposition.^31,32^ As a result, consistently identifying patients and transforming clinical practice may be difficult.

We did identify a significant difference in in-hospital mortality, which includes those who died in GIP hospice. Hospitals are measured on inpatient mortality rates as a function of hospital quality.^33^ Patients admitted to hospice are excluded from observed mortality in most publicly reported metrics. Studies have shown that increased hospice use is associated with decreased inpatient hospital mortality.^34^ Some have criticized this approach to measuring inpatient mortality as being susceptible to “hospice flipping,” in which patients are admitted to GIP hospice when death is imminent without the patient and family receiving hospice benefits.^35^ We espouse the development of programs such as ED CTP that instead seek to identify patients early in the course of presentation based on goals of care discussions and leverage efficient workflows to expedite hospice transitions to maximize benefits and ensure patient-centered, timely care.

Hospitals and ED clinicians may be motivated by published data that show that early hospice transition can reduce hospital LOS, improving both inpatient and ED crowding at capacity-constrained hospitals.^14^ However, we found no significant difference in hospital or ED LOS for the overall cohort. This finding could be a result of our identification strategy, which was limited to 96 hours from presentation, or workflow inefficiencies that need to be streamlined.

There are several areas for future research on transitions from the ED to hospice. For example, ongoing efforts to create and sustain educational campaigns to influence the perception of hospice as patient and family centered are needed. There is also a need to increase the frequency and effectiveness of serious illness conversations to better memorialize and communicate goals of care. Furthermore, novel pathways, such as palliative extubation in the ED, should be developed and studied.^36^ Similarly, ED observation unit protocols for a comfort-focused transition to hospice should be explored; this setting has previously been used to allow for the direct placement of patients into skilled nursing facilities.^37^ Finally, implementation science efforts that use validated metrics to track improvement initiatives over time and cross-hospital comparisons would be helpful.

### Limitations

Our study had several limitations. First, it was implemented at a single health care center, so results may have limited generalizability, particularly for hospitals with limited care coordination resources and relationships with hospice vendors. Hospice use could be partly influenced by secular trends as opposed to program implementation. Looking at all hospice transitions in our hospital over the past 5 years illustrates a sustained increase in third quarter of 2022 during the intervention period (eFigure 3 in Supplement 1). This timing also corresponded with the addition of an additional GIP hospice vendor (approximately 70% of GIP hospice volume during this time period). The close affiliation between our institution and the Dana-Farber Cancer Institute allowed us to have more statistical power for the subgroup analysis on cancer patients, but it may make our data nonrepresentative of other ED facilities. We were also unable to consistently determine the precise date of death among patients discharged from the hospital, with or without hospice care; this limitation precluded our ability to examine potentially important outcomes, such as time from ED visit to death among different patient groups. Second, we relied on expertise from multiple content areas to design and implement our program. In addition, digital tools for patient screening may not be widely available. Other centers will need multistakeholder input and adaptation to the specific workflows of their institution. Third, our study design did not allow for a defined concurrent control group, which raises the concern about differences in hospice care. Fourth, our data and outcomes were limited to operational variables; we aim to add data about patient and family experiences.

## Conclusions

In our quality improvement study, a multidisciplinary program to transition patients near the end of life to hospice was associated with a 2.4-fold increase in goal-concordant hospice care without requiring inpatient hospital admission. Further investigation is needed to examine generalizability and reexamine results for sustainability over time.

## Notes

Supplement 1.
eMethods.
**eFigure 1.** Emergency Department (ED) Care Transitions Program Workflow **eFigure 2.** ED CTP Reference Guide **eFigure 3.** Overall Hospital Hospice Enrollment Trend **eTable 1.** Characteristics of Study Subjects With Cancer **eTable 2.** Characteristics of Study Subjects With Neurological Diagnoses **eTable 3.** For Patients With Cancer, Multivariate Logistic Regression Model for Primary Outcome of Patient Transitioning From the ED to Hospice Within 96 Hours Without Admission **eTable 4.** For Patients With Neurologic Diagnoses, Multivariate Logistic Regression Model for Primary Outcome of Patient Transitioning From the ED to Hospice Within 96 Hours Without Admission

Supplement 2.
Data Sharing Statement
