# Supplementary material for: A Hospice Transitions Program for Patients in the Emergency Department
Source: JAMA Netw Open. 2024 Jul 8;7(7):e2420695. doi: 10.1001/jamanetworkopen.2024.20695 (PMC11231795; doi:10.1001/jamanetworkopen.2024.20695)
Supplement: Supplement 1. — eMethods. eFigure 1. Emergency Department (ED) Care Transitions Program Workflow eFigure 2. ED CTP Reference Guide eFigure 3. Overall Hospital Hospice Enrollment Trend eTable 1. Characteristics of Study Subjects With Cancer eTable 2. Characteristics of Study Subjects With Neurological Diagnoses eTable 3. For Patients With Cancer, Multivariate Logistic Regression Model for Primary Outcome of Patient Transitioning From the ED to Hospice Within 96 Hours Without Admission eTable 4. For Patients With Neurologic Diagnoses, Multivariate Logistic Regression Model for Primary Outcome of Patient Transitioning From the ED to Hospice Within 96 Hours Without Admission [file jamanetwopen-e2420695-s001.pdf]

Supplementary Online Content

Baugh CW, Ouchi K, Bowman JK, et al. A hospice transitions program for patients in the emergency department. *JAMA Netw Open.* 2024;7(7):e2420695.
doi:10.1001/jamanetworkopen.2024.20695

eMethods.
eFigure 1. Emergency Department (ED) Care Transitions Program Workflow
eFigure 2. ED CTP Reference Guide
eFigure 3. Overall Hospital Hospice Enrollment Trend
eTable 1. Characteristics of Study Subjects With Cancer
eTable 2. Characteristics of Study Subjects With Neurological Diagnoses
eTable 3. For Patients With Cancer, Multivariate Logistic Regression Model for Primary Outcome of Patient Transitioning From the ED to Hospice Within 96 Hours Without Admission
eTable 4. For Patients With Neurologic Diagnoses, Multivariate Logistic Regression Model for Primary Outcome of Patient Transitioning From the ED to Hospice Within 96 hours Without Admission

This supplementary material has been provided by the authors to give readers additional information about their work.

© 2024 Baugh CW et al. *JAMA Network Open.*

eMethods.

Screening and Identification

Screening for potential candidates occurs via natural language processing via Admit-Discharge-Transfer Health Level Seven (ADT HL7) feeds and case-by-case screening by clinical staff. Natural language processing code was written de novo by our information services "data lake team." The code is a simple string-matching approach using either exact phrases or regular expressions to identify diagnosis codes attached to the ADT HL7 feed. Results are refreshed every 15 minutes and alerts are sent to emergency department (ED) care facilitator staff via automated email for patients with neurological or cancer diagnoses. ED care facilitators are expected to review each email alert during their shift and reply with patient eligibility. The full code may be requested from our data lake team.

Individual case screening was interdisciplinary, largely led by ED clinicians and care facilitators. All clinical teams were educated to identify patients who presented from hospice or are likely to transition to hospice within 96 hours as potential pathway patients. These general criteria are intentionally left broad to allow for clinical correlation on a case-by-case basis. All patients identified as potential candidates are sent to the ED care facilitator for formal screening, which is a key component of the intervention. If eligible for the pathway, the ED care facilitator will alert the admitting team to the appropriate level of care and discuss whether hospice referrals are appropriate. If not eligible, the patients receive routine care, including palliative care services as appropriate. The ED care facilitators document their screening summary in a note template in the electronic health record (EHR) consisting of a brief patient history, whether they are eligible for the pathway, and whether hospice referrals are placed.

© 2024 Baugh CW et al. *JAMA Network Open.*

Modified Workflows and Education

Provider workflows, initially a barrier to hospice utilization due to a cumbersome EHR-based admission process, were a focus of improvement. Working with admitting, compliance, medical records, revenue cycle, and information services, we streamlined the EHR-based admission process. Furthermore, the revenue cycle team reviews each hospice pathway enrolled patient to ensure that there is no impact on out-of-pocket patient costs.

Education is a key driver of pathway performance. Over twenty interprofessional clinical teams are educated at least biannually by operational leads (ML and LMO); these teams include ED nurses, advanced practice providers, attendings, trainees, nurse care coordinators, specialty clinicians (attendings, advanced practice providers, trainees), palliative care and nursing staff. Education sessions are approximately 15 minutes long, typically at least twice a year, and consist of an overview of hospice and services offered, pathway workflow logistics, patient feedback and experience data, and case examples. Education is reinforced continuously via workflow graphics and tip sheets created to meet the needs of various provider settings and role groups. Printed copies are posted in key work areas and contain QR codes to digital tip sheets. A patient-facing brochure providing an overview of hospice and the services available was created in partnership with a multidisciplinary team, including a patient-family advocate, hospital staff, and hospice vendors.

Data Tracking and Reporting

At the time of program implementation, reports were created to capture all patients who were discharged to hospice or expired in the hospital. These reports help facilitate weekly case reviews with interprofessional pathway stakeholders, including utilization management, ED care

© 2024 Baugh CW et al. *JAMA Network Open.*

facilitators and inpatient case managers, case management leadership, palliative care, frontline clinical champions (neuroscience and oncology medical leadership), and operational leaders. At each weekly review meeting, all reported cases are classified as eligible and hospice pathway enrolled, eligible and hospice pathway not enrolled, or ineligible and recorded on a master patient tracker that allows for graphical representations of progress and an ability to identify trends quickly. Providers caring for patients who were deemed eligible and hospice pathway not enrolled receive email feedback, which has been helpful in identifying barriers to pathway utilization or educational gaps. Weekly enrollment reports are sent to pathway steering committee members, consisting of key institutional stakeholders and divisional and executive leadership. Steering committee meetings are held monthly to provide updates on progress and identify barriers.

New Hospice Vendor Relationships

When launching the program, new hospice vendor relationships were pursued to expand access to patients seeking hospice services. At the time of contracting, mutually agreed-upon performance metrics were created for each vendor, including time to patient admission and length of stay. Hospice note templates were created in alignment with both vendors to standardize the documentation process in our EHR, allowing for improved communication. The operational leads meet monthly with each vendor to identify successes and opportunities.

© 2024 Baugh CW et al. *JAMA Network Open.*

eFigure 1. Emergency Department (ED) Care Transitions Program Workflow

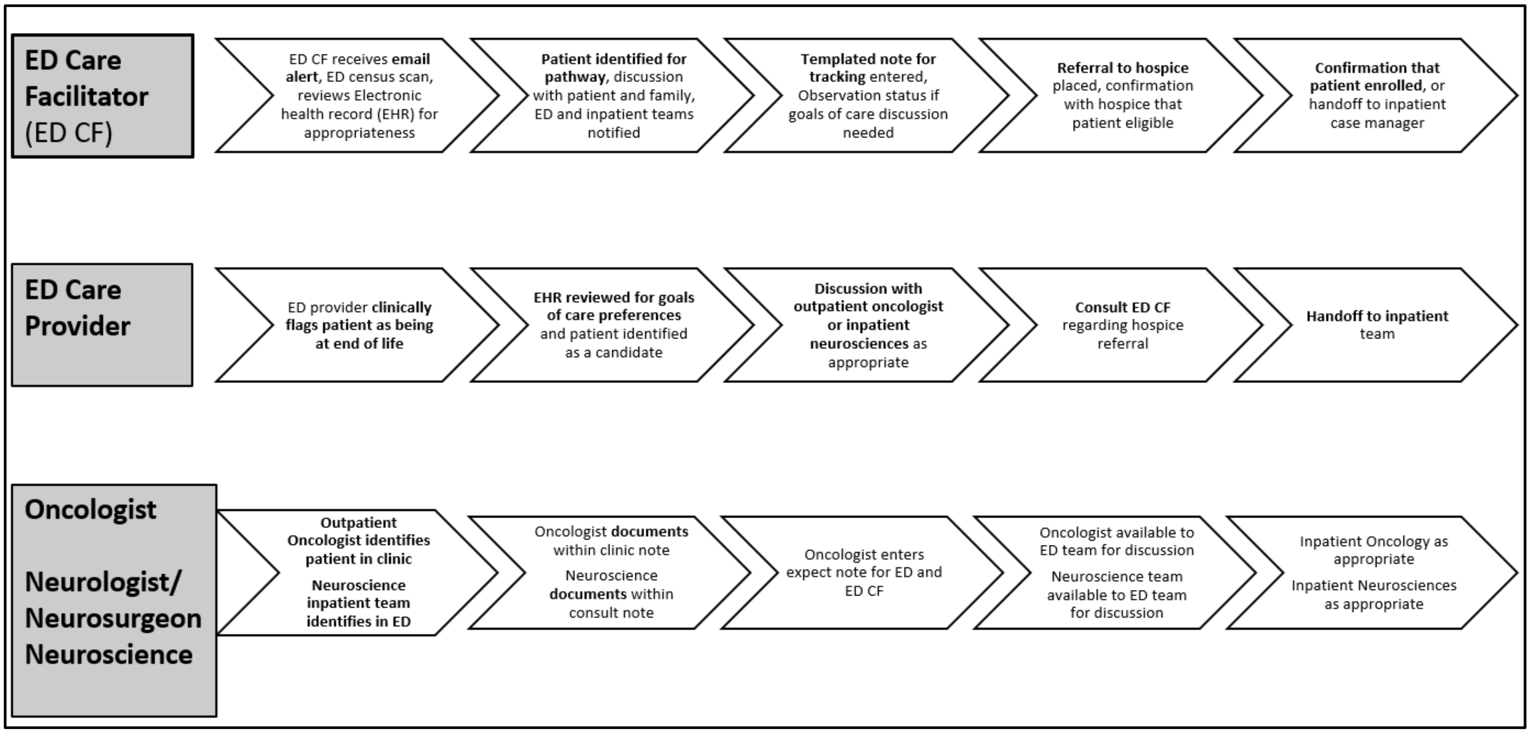

Abbreviations: ED (Emergency Department); EHR (Electronic Health Record)

© 2024 Baugh CW et al. *JAMA Network Open.*

eFigure 2. ED CTP Reference Guide

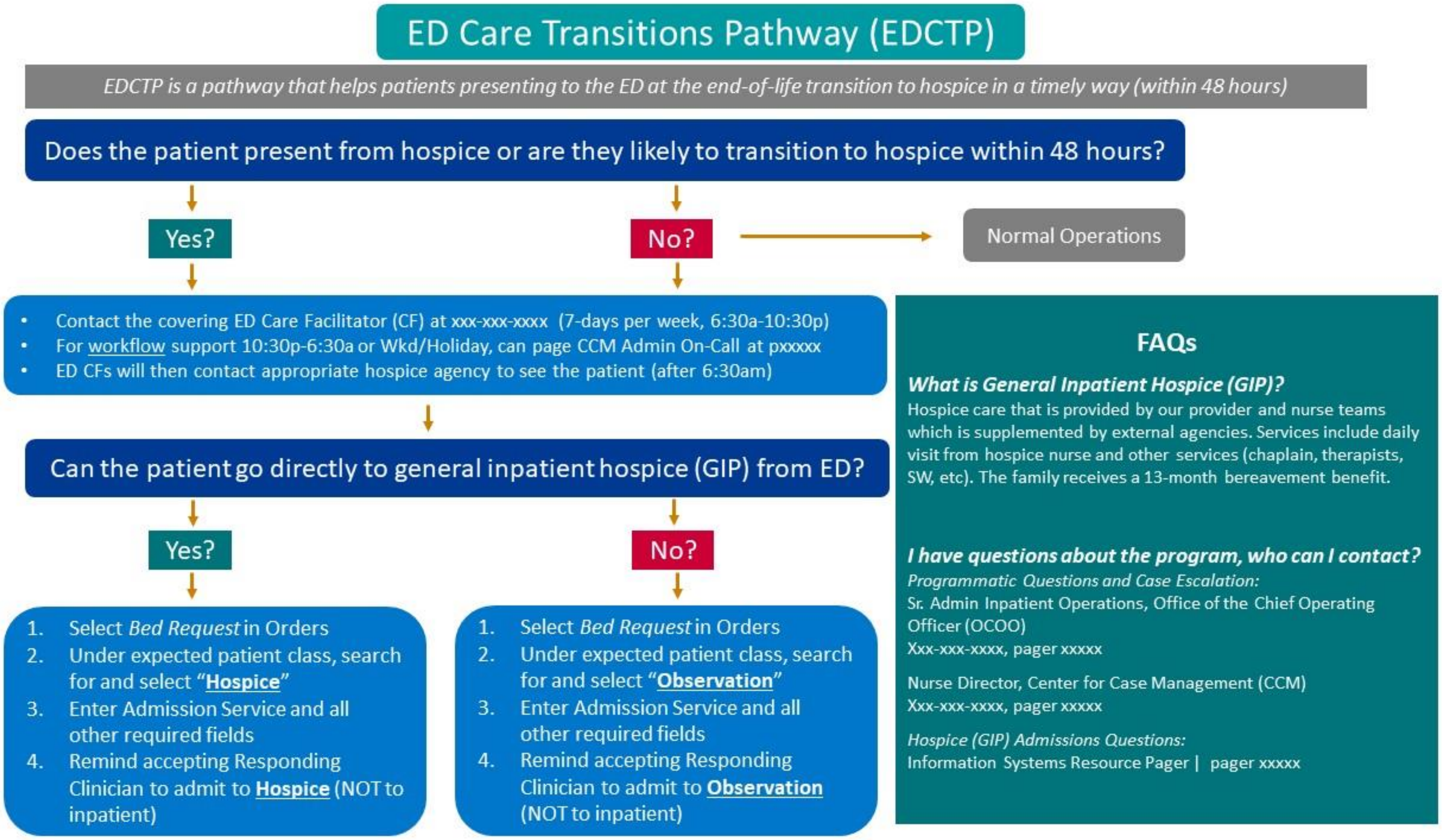

Abbreviations: ED (Emergency Department); WKD (Weekend); CCM (Center for Case Management); FAQ (Frequently Asked Questions)

© 2024 Baugh CW et al. *JAMA Network Open.*

eFigure 3. Overall Hospital Hospice Enrollment Trend

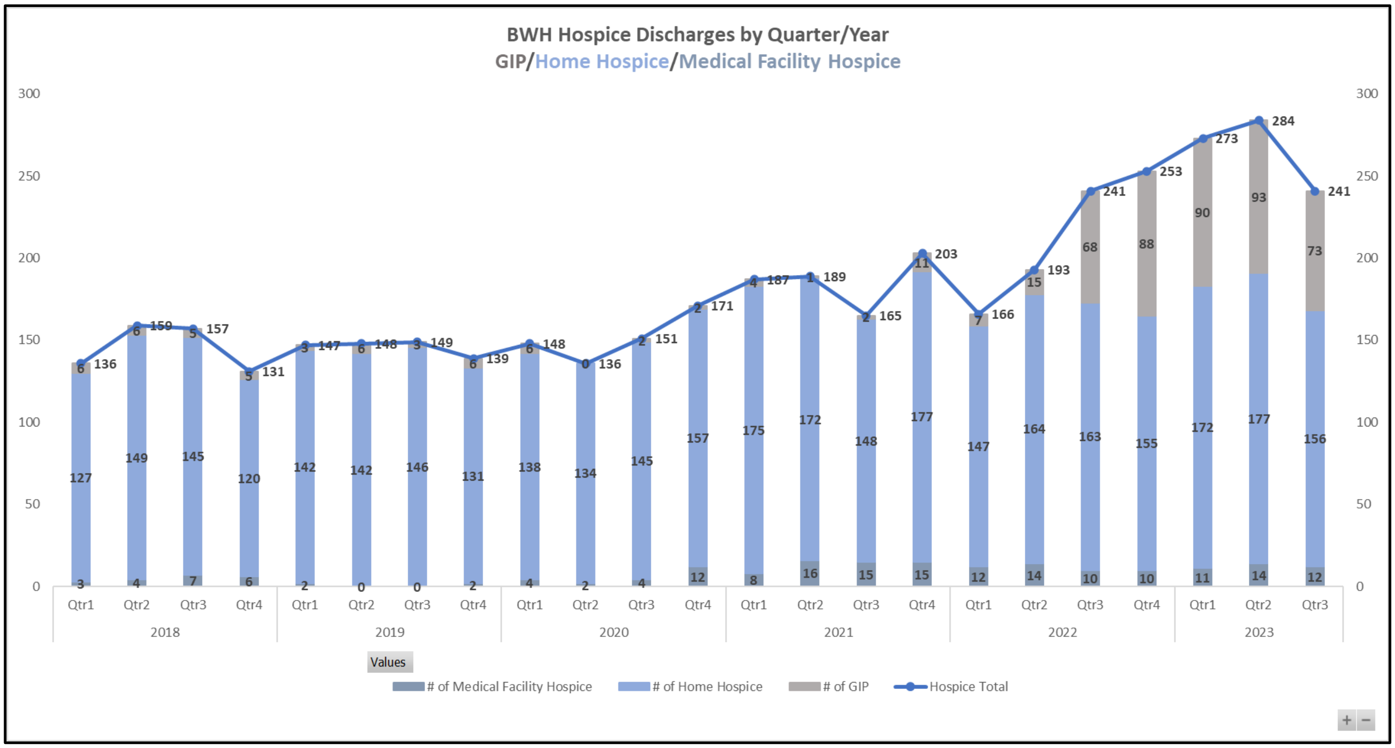

Across all services, inclusive of all admission status and length of stay (not limited to 96 hours)

© 2024 Baugh CW et al. *JAMA Network Open.*

eTable 1. Characteristics of Study Subjects With Cancer

Patient Characteristic	Control	Intervention	P-value
Number in cohort	61	134	
Age median (IQR) in years	69.0 (61.0-75.0)	66.0 (56.0-77.0)	0.86
Sex (n, %)			0.43
Male	28 (45.9%)	53 (39.6%)	
Female	33 (54.1%)	81 (60.5%)	
Cancer Type (n, %)			
Solid Tumor	49 (80.3%)	102 (76.1%)	0.53
Hematologic	2 (3.3%)	10 (7.4%)	
Solid cancer stage			
1-3	4 (6.56%)	7 (5.23%)	
4	42 (68.9%)	79 (59.0%)	0.58
Race (n, %)			0.43
White	48 (78.7%)	97 (72.4%)	
Black	6 (9.8%)	11 (8.2%)	
Asian	2 (3.3%)	11 (8.2%)	
Other	2 (3.3%)	12 (9.0%)	
Ethnicity (n, %)			0.60
Hispanic	7 (11.5%)	11 (8.2%)	
Non-Hispanic	54 (88.5%)	120 (89.6%)	
Language (n, %)			0.36
English	56 (91.8%)	113 (84.3%)	
Spanish	1 (1.6%)	4 (3.0%)	
Other	4 (6.6%)	17 (12.7%)	
Education (n, %)			0.80
Graduate School	6 (9.8%)	20 (14.9%)	
College	25 (41.0%)	56 (41.8%)	
High School	23 (37.7%)	42 (31.3%)	
Less than High School	5 (8.2%)	8 (6.0%)	
Other	2 (3.3%)	5 (3.7%)	
Primary Payer (n, %)			0.72
Medicare	27 (44.3%)	50 (37.3%)	
Commercial	31 (50.8%)	77 (57.5%)	
Medicaid	2 (3.3%)	6 (4.5%)	
Other	1 (1.6%)	1 (0.8%)	

© 2024 Baugh CW et al. *JAMA Network Open.*

Documentation of Prior Serious Illness Conversation (n, %)	4 (6.6%)	31 (23.1%)	0.005
Presence of MOLST at the time of ED arrival (n, %)	25 (41.0%)	74 (55.2%)	0.09
Charlson Comorbidity Score median (IQR)	3.0 (2.0-5.0)	3.0 (2.0-4.0)	0.43
ED median length of stay (IQR) in hours	4.23 (1.63-6.57)	4.68 (0-6.84)	0.65
Hospital LOS median (IQR) in days	2.20 (1.88-2.89)	2.24 (1.79-3.45)	0.53
In-hospital mortality	21 (34.4%)	28 (20.9%)	0.05

Abbreviations: ED, Emergency Department; MOLST (Medical Orders for Life-Sustaining Treatment); LOS (length of stay)
Missing or unidentified data from electronic health records: Race (n=6), Ethnicity (n=3), Education (n=3), Cancer Type (n=31), MOLST (n=29) Percentage denominators presented include total control (61) and intervention (134), inclusive of missing data.

© 2024 Baugh CW et al. *JAMA Network Open.*

eTable 2. Characteristics of Study Subjects With Neurological Diagnoses

Patient Characteristics	Control	Intervention	P-value
Number in cohort	56	71	
Age median (IQR) in years	82.5 (71.0-87.0)	80.0 (69.0-86.0)	0.93
Sex (n, %)			1.00
Male	27 (48.2%)	35 (49.2%)	
Female	27 (48.2%)	35 (49.2%)	
Race (n, %)			0.33
White	40 (71.4%)	60 (84.5%)	
Black	1 (1.8%)	3 (4.2%)	
Asian	2 (3.6%)	0 (0%)	
Other	1 (1.8%)	1 (1.4%)	
Ethnicity (n, %)			1.00
Hispanic	2 (3.6%)	4 (5.6%)	
Non-Hispanic	38 (67.9%)	59 (83.1%)	
Language (n,%)			0.49
English	43 (76.8%)	64 (90.1%)	
Spanish	1 (1.8%)	1 (1.4%)	
Other	1 (1.8%)	5 (7.0%)	
Education (n, %)			0.13
Graduate School	1 (1.8%)	6 (8.5%)	
College	18 (32.1%)	17 (23.9%)	
High School	10 (17.9%)	26 (36.6%)	
Less than High School	4 (7.1%)	10 (14.1%)	
Other	0 (0%)	1 (1.4%)	
Primary Payer (n, %)			0.20
Medicare	31 (55.4%)	46 (64.8%)	
Commercial	18 (32.1%)	23 (32.4%)	
Medicaid	3 (5.4%)	1 (1.4%)	
Other	4 (7.1%)	1 (1.4%)	
Documentation of Prior Serious Illness Conversation (n, %)	2 (3.6%)	7 (9.9%)	0.30
Presence of MOLST at the time of ED arrival (n, %)	6 (10.7%)	25 (35.2%)	0.003
Charlson Comorbidity Score median (IQR)	4.0 (3.0-5.0)	4.0 (3.0-5.0)	0.70
ED median length of stay (IQR) in hours	2.36 (1.58-3.70)	4.30 (2.16-6.07)	0.003
Hospital LOS median (IQR) in days	1.87 (1.16-2.99)	1.97 (0.96-2.85)	0.70

© 2024 Baugh CW et al. *JAMA Network Open.*

In-hospital mortality	47 (83.9%)	35 (49.3%)	<0.001

Abbreviations: ED, Emergency Department; MOLST (Medical Orders for Life-Sustaining Treatment); LOS (length of stay)
Missing or unassigned data from electronic health records: Sex (n=3), Race (n=19), Ethnicity (n=24), Language (n=12), Education (n=35), MOLST (n=41). Percentage denominators presented include total control (56) and intervention (71), inclusive of missing data.

© 2024 Baugh CW et al. *JAMA Network Open.*

eTable 3. For Patients with Cancer, Multivariate Logistic Regression Model for Primary Outcome of Patient Transitioning from the ED to Hospice Within 96 Hours Without Admission

Characteristic	Adjusted Odds Ratio* (95% CI)
**Intervention** (Control=reference)	
	4.19 (2.04-8.60)
**Demographics**	
Age ≥65 (<65=ref)	0.98 (0.95-1.02)
**Race** (White=reference)	
Black	1.58 (0.42-5.91)
Asian	1.42 (0.29-6.89)
Other	1.24 (0.30-5.03)
**Insurance** (Commercial=reference)	
Medicare	1.10 (0.49-2.51)
Medicaid	0.68 (0.07-6.66)
Other	0.72 (0.04-13.18)
**MOLST** (No MOLST=reference)	
	2.08 (1.02-4.26)
**Charlson comorbidity index**	1.11 (0.91-1.36)

Abbreviations: MOLST (Medical Orders for Life-Sustaining Treatment)

© 2024 Baugh CW et al. *JAMA Network Open.*

eTable 4. For Patients with Neurologic Diagnoses, Multivariate Logistic Regression Model for Primary Outcome of Patient Transitioning from the ED to Hospice Within 96 Hours Without Admission

Characteristic	Adjusted Odds Ratio* (95% CI)
**Intervention** (Control=reference)	
	2.05 (0.78-5.37)
**Demographics**	
Age ≥65 (<65=ref)	0.98 (0.92-1.04)
**Race** (non-White=reference)	
White	3.52 (1.09-11.31)
**Insurance** (Commercial=reference)	
Medicare	0.55 (0.17-1.73)
Medicaid	0.31 (0.02-5.65)
Other	0.67 (0.05-9.79)
**MOLST** (No MOLST=reference)	
	4.36 (1.22-15.55)
**Charlson comorbidity index**	1.28 (0.64-2.55)

Abbreviations: MOLST (Medical Orders for Life-Sustaining Treatment)

© 2024 Baugh CW et al. *JAMA Network Open.*
