# Supplementary material for: A Hospice Transitions Program for Patients in the Emergency Department
Source: JAMA Netw Open. 2024 Jul 8;7(7):e2420695. doi: 10.1001/jamanetworkopen.2024.20695 (PMC11231795; doi:10.1001/jamanetworkopen.2024.20695)
Supplement: Supplement 2. — Data Sharing Statement [file jamanetwopen-e2420695-s002.pdf]

Data Sharing Statement

Baugh. A Hospice Transitions Program for Patients in the Emergency Department. *JAMA Netw Open*. Published July 08, 2024. doi:10.1001/jamanetworkopen.2024.20695

Data
Data available: No

Additional Information
Explanation for why data not available: We can offer a data dictionary but cannot share patient level data that risks patient privacy such as PHI elements
